# Small molecules increase direct neural conversion of human fibroblasts

**DOI:** 10.1038/srep38290

**Published:** 2016-12-05

**Authors:** Ulrich Pfisterer, Fredrik Ek, Stefan Lang, Shamit Soneji, Roger Olsson, Malin Parmar

**Affiliations:** 1 Department of Experimental Medical Science, Wallenberg Neuroscience Center 221 84, Lund, Sweden; 2 Lund Stem Cell Center, Lund University, 221 84, Lund, Sweden; 3 Chemical Biology & Therapeutics, Department of Experimental Medical Science, Lund University, 221 84, Lund, Sweden; 4 Division of Molecular Hematology, BMC, Lund University, Lund 22242, Sweden

## Abstract

The generation of human induced neurons (hiNs) via exogenous delivery of neural transcription factors represents a novel technique to obtain disease and patient specific neurons. These cells have the potential to be used for disease modeling, diagnostics and drug screening, and also to be further developed for brain repair. In the present study, we utilized hiNs to develop an unbiased screening assay for small molecules that increase the conversion efficiency. Using this assay, we screened 307 compounds from five annotated libraries and identified six compounds that were very potent in potentiating the reprogramming process. When combined in an optimal combination and dose, these compounds increased the reprogramming efficiency of human fibroblasts more than 6-fold. Global gene expression and CellNet analysis at different timepoints during the reprogramming process revealed that neuron-specific genes and gene regulatory networks (GRNs) became progressively more activated while converting cells shut down fibroblast-specific GRNs. Further bioinformatics analysis revealed that the addition of the six compound resulted in the accelerated upregulation of a subset of neuronal genes, and also increased expression of genes associated with transcriptional activity and mediation of cellular stress response.

Somatic cell reprogramming using defined transcription factors enables the generation of induced pluripotent stem (iPS) cells^1^,^2^,^3^, as well as allows for the direct conversion of somatic cells into terminally differentiated cells, including subtype-specific and functional neurons^4^,^5^,^6^,^7^,^8^,^9^,^10^. A number of studies have shown that addition of small molecules during re-programming into pluripotency^11^,^12^,^13^,^14^ or during direct cell fate conversion^15^,^16^ increase the efficiency and/or survival^17^, and in some cases allow for chemical replacement of individual reprogramming genes^18^,^19^,^20^ or even completely replace the need for transgene expression^21^. Candidate approaches have so far identified a number of SMs (dual SMAD inhibition and Forskolin) that can potentiate neural conversion of human fibroblasts^22^,^23^ and proof of principle that human fibroblasts and glia can be converted into iNs using only addition of defined combinations of chemical compounds have recently been reported^24^,^25^,^26^.

Here, we report the development of an unbiased automated assay to identify SMs enhancing direct neuronal conversion into human induced neurons (hiNs). We focused the screen on annotated libraries (Kinase inhibitors, Epigenetic modulators, Wnt pathway, Nuclear receptors and Phosphatase inhibitors) with compounds that have a reported effect on pathways and target proteins known to be involved in cell maturation, growth and survival. By screening >300 compounds, we identified 20 compounds (5 epigenetic regulators, 8 kinase inhibitors, 5 wnt regulators, 2 nuclear receptor ligands) that increase efficiency and purity of direct neuronal reprogramming of human fibroblasts. Based on dose escalation studies, we selected 6 compounds that increased conversion efficiency in an optimal concentration range that was significantly different from the toxic dose: the Gsk3β inhibitor kenpaullone, cAMP/PKA modulator prostaglandin E2 (PGE2), adenylyl cyclase activator forskolin, HDAC inhibitor BML210, SIRT1 activator aminoresveratolsulfat and Src kinase inhibitor PP2. The small molecules identified in the present study differ from compounds previously described in neuronal reprogramming, however they target, at least in part, similar signaling pathways.

In order to gain a better understanding of how these compounds acted during the early stages of reprogramming, we preformed a global gene expression analysis of FACS purified hiNs obtained in the absence and presence of the compounds. We first performed CellNet analysis, which classifies cells using a large body of publicly available data (29, 30), which revealed initiation of neuron- specific gene regulatory networks (GRNs) as well as ablation of fibroblasts- specific GRNs, which occurred at similar rates among all groups of converting hiNs. Further bioinformatics analysis of this time course experiment enabled for a more detailed view on transcriptional changes and revealed that the addition of the six compounds resulted in the accelerated upregulation of a subset of neuronal genes, and also increased expression of genes associated with transcriptional activity and mediation of cellular stress response early during the reprogramming process.

## Results

**Figure 1:**
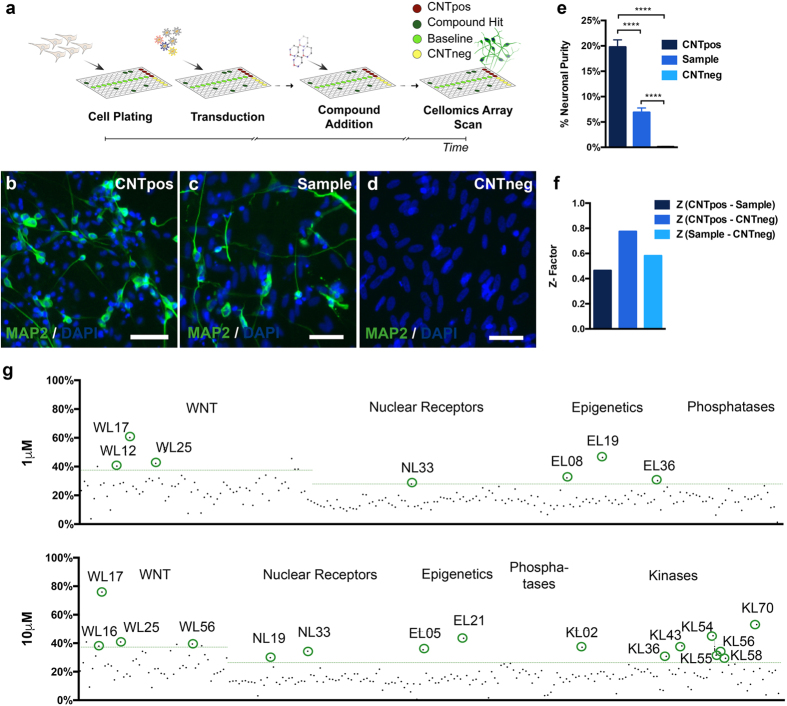
Assay development for high- content screening of small molecules using human induced neurons. **(a)** Schematic representation of the experimental outline using hiNs for small molecule screening. (**b–d**) Conditions defining the borders of the screening assay. (**b**) CNTpos: ABM- converted + small molecules (SMs). (**c**) Sample condition: ABM- converted+/− compounds to be tested. (**d**) Untransduced cells, no SMs, no compounds to be tested (Scale bars 50 μm). (**e**) Cellomics array quantifications of neuronal purity in the different assay conditions. All conditions are significantly different from each other (Alpha level p < 0.001, one- way ANOVA with Tukey post hoc test). (**f**) Z- Factor calculation to determine the screening window between the different conditions. (**g**) Representation of the neuronal purity relative to the CNTpos induced by single tested compounds. Single compounds from all libraries (except kinase library) were tested at concentrations 1 μM and 10 μM. Dotted green lines representing the 3xSD distance from the average sample condition without compounds (significance threshold). Green Circles indicating primary hit compounds selected based on both the induction of a high relative neuronal purity and a neuronal morphology.

We first developed an unbiased assay amenable for high-content screening of SMs which increase neuronal induction, assessed by using automated cell counting of MAP2^+^ cells as a primary readout (Fig. 1a). As positive control (CNTpos) fibroblasts were converted using a transcription-factor based protocol modified from^22^ and that robustly yields high conversion efficiency and purity^27^ (Fig. 1b), transcription-factor-only converted fibroblasts were used to define the actual sample condition used to test individual compounds (Fig. 1c), and unconverted fibroblasts served as negative control (CNTneg) (Fig. 1d).

Automated quantification of MAP2^+^ hiNs performed on day 12 after transgene expression using Cellomics array scan (Array Scan VTI, Thermo Fischer) yielded a neuronal purity of 19.75 ± 1.41 (CNTpos, n = 29), 6.88 ± 0.88 (sample, n = 31) and 0.11 ± 0.06 (CNTneg, n = 31) (Fig. 1e). Z- Factors (CNTpos- Sample: 0.46; CNTpos- CNTneg: 0.77; Sample- CNTneg: 0.58) (Fig. 1f) indicate that the different assay conditions are sufficiently separated from each other to detect putative hits (Zhang *et al*.)^28^.

Using this assay, we subsequently screened >300 small molecules from five annotated libraries, containing compounds that target the Wnt-signaling pathway and nuclear receptors, as well as epigenetic regulators, phosphatase and kinase inhibitors (Enzo Life science). Compounds from all libraries were screened at concentrations of 1 μM and 10 μM except kinase library, KL, which was screened at 10 μM only. The resulting neuronal purity for each compound was presented relative to the CNTpos (Fig. 1g). Positive hits were identified based on the induction of a relative neuronal purity above the set threshold (average sample + 3xSD, dotted green line) and the absence of obvious cell toxicity based on number of dapi counts (not shown). In total, 20 small molecules were selected as primary hits for increasing the neuronal purity above the set threshold (Fig. S1 and Table S1), and one additional compound was selected based on its ability to induce a mature neuronal morphology (EL38). It was noteworthy to observe that some of the compounds implicated in hiN conversion, such as CHIR99021, valproic acid and ROCK inhibitor Y-27632 appeared in the screen, but at sub-threshold levels.

**Figure 2:**
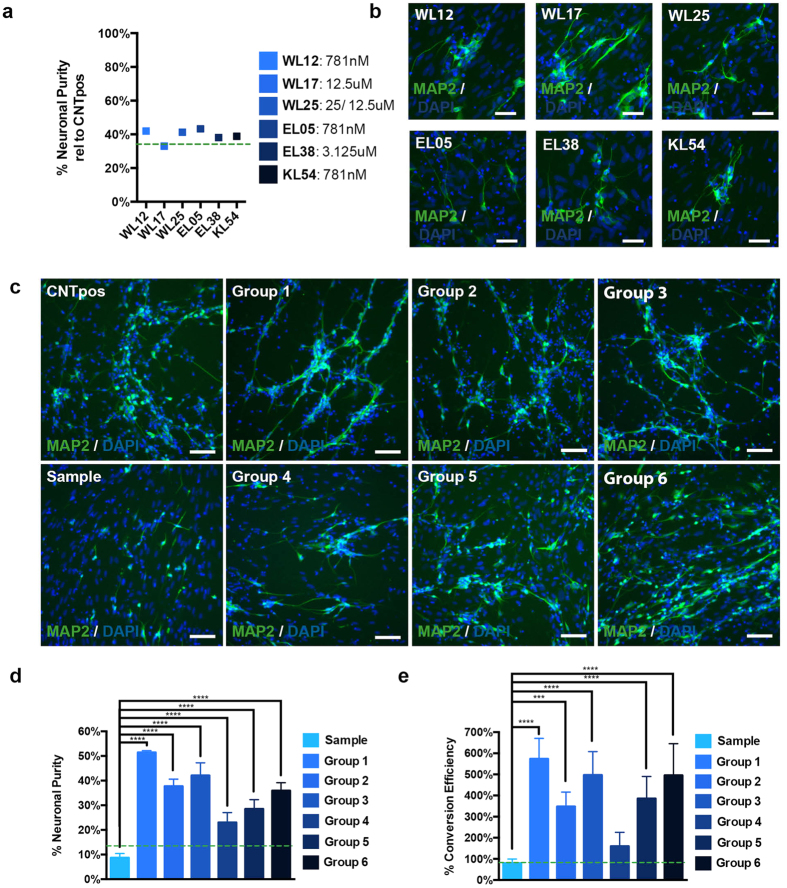
Confirmation of hit compounds and application of compound hits in selected groups. (**a**) Maximum relative neuronal purity achieved by identified hit compouds and corresponding concentration. (**b**) Representative images of identified final 6 compounds. (**c**) Representative images of the assay conditions CNTpos and sample as well as of six different conditions using selected groups of compounds (see Table S2B). (**d**) Neuronal Purity yielded by converting hiNs in the presence of different compound combinations. All compound groups let to a strong increase in neuronal purity compared to the sample condition. (**e**) Conversion efficiency in the presence of different groups of compounds. Except for compound group 4, all other groups yielded significantly increased conversion efficiency compared to the sample condition. (Alpha level p < 0.05, one- way ANOVA with Bonferroni post hoc test) (Dotted green line: average sample + 3xSD) (Scale bars: 100 μm).

In order to validate primary hits, selected compounds were re-tested in a dose-response experiment. An increasing concentration range from 48.8 nM – 50 μM was applied for each compound and compounds were determined as confirmed hits if they induced a relative neuronal purity above the set threshold (average sample +3xSD, dotted green line) in a concentration-dependent manner, before reaching cytotoxicity (grey area). Thus, 11 of the 21 hits were confirmed (Fig. S2 a–k). Interestingly, several compounds individually reached up to 60% of the neuronal purity of the CNTpos (Fig. S2c,e,g,h and k). Next, we re-tested all 11 secondary hits from dry powder in a dose- response experiment, and six final compounds were validated to significantly increase relative neuronal purity (Fig. 2a,b and S3 a–f). This also yielded more refined concentration curves and a more accurate concentration optimum for each compound.

Next, we wanted to explore combinatorial effects of the selected compounds to further improve direct neuronal conversion. We therefore treated cells of the sample condition with different combinations of SMs using the concentration optima established previously (Table S2a). Group 1 combines all identified compounds and evaluates their synergistic effects on neuronal conversion. Group 2 combines novel six compounds together with small molecules and growth factors from CNTpos to identify whether stimulation of multiple signaling of both conditions would be most beneficial for conversion. Group 3 combines increased cAMP signaling and Gsk3β inhibition mediated by three of the novel screened compounds. This group evaluates whether stimulation of this signaling provides the most crucial contribution to increased conversion. Increased cAMP signaling and Gsk3β inhibition has previously been shown to enhance neuronal conversion (Ladewig^22^; Li^23^). Group 4 in contrast evaluates the potency of Src kinase, HDAC inhibition and SIRT activation alone since this signaling has not been associated previously with direct neuronal reprogramming. In order to futher test the contribution of different compounds and to reduce combinatorial space, Group 5 and 6 represent combinations of the six novel compounds based on a best guess approach to evaluate either SIRT1 activation together with stimulated cAMP signaling/Gsk3β inhibition (Group 5) or stimulated cAMP signaling/Gsk3β inhibition together with the epigenetic regulator/HDAC inhibitor BML210 (Table S2b).

All different compound conditions resulted in the induction of MAP2 expressing hiNs with neuronal morphology (Fig. 2c) and all combinations of compounds passed the threshold for both relative neuronal purity as well as conversion efficiency (average sample +3xSD) (Fig. 2d,e, dotted green line). Combining all six hit compounds yielded a neuronal purity of 50% and almost 500% conversion efficiency (Fig. 2d,e), while the combination of compounds of group 4 yielded the lowest increase in neuronal purity and conversion efficiency (Fig. 2d,e).

To gain insight at the molecular level how these compounds act during the reprogramming process, we performed global gene expression analysis of FACS sorted hiNs obtained under the following conditions: baseline, CNTpos, forskolin alone and 6 compounds. In order to FACS sort converting hiNs, we linked the expression of Ascl1 to expression of a fluorescent reporter^27^. To achieve temporal resolution of the effects of different treatments, converted cells were analysed after the following days of transgene expression: day 2 (=0 days exposure to small molecules, ESM), day 5 (=2 days ESM), day 7 (=4 days ESM) and day12 (=9 days ESM). Unconverted fibroblasts were used as reference control.

**Figure 3:**
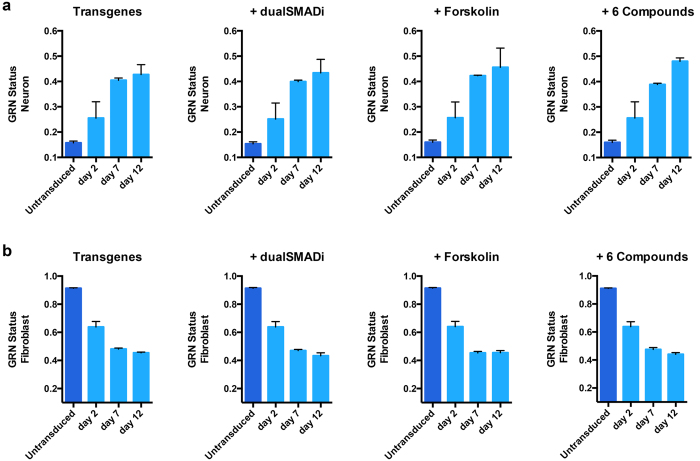
CellNet analysis for gene regulatory networks. (**a**) Increase of gene regulatory network (GRN) score for neurons with increased reprogramming time across all experimental groups. (**b**) Simultaneous decrease of fibroblast GRN score with increased reprogramming time across all experimental groups.

First, we examined the emergence of a global neuronal fate using the newly established online platform CellNet^29^,^30^. This analysis shows that all groups revealed a similar decline in fibroblast- specific GRNs while activating neuron- specific gene regulatory networks (GRNs) to the same extend along the time course (Fig. 3a,b). The sequencing also allowed us to confirm that correct target genes of each pathway was up and down regulated in the 6 compound group, confirming activation of the corresponding pathways (Fig. S3g).

**Figure 4:**
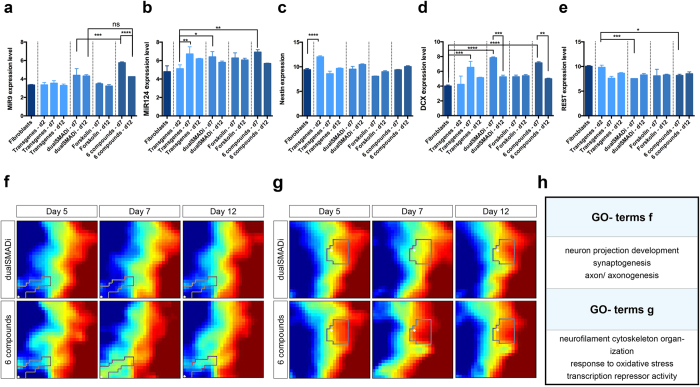
Gene expression analysis using micro array. (**a–e**) Expression of identified candidate genes MiR9, MiR124, Nestin, DCX and REST and alteration of expression level in different experimental groups and at different experimental stages. (**a**) Transient increase in expression of the known driver of neuronal conversion MiR9 at day 7 of conversion in dualSMADi and 6 compound condition with 6 compound condition yielding highest MiR9 levels at day 7. (**b**) Intermediate increase in expression of MiR124 at day 7 of conversion. (**c**) Two days of transgene expression induces transient increase in Nestin expression indicative of a transient progenitor state. In line with this, except for forskolin condition, all other experimental groups revealed increased expression levels of DCX at day 7 of conversion. (**e**) Intermediate reduction of REST expression in both day 7 time points of dualSMADi and 6 compound groups. (**f–h**) Gene expression dynamics inspector (GEDI) analysis using micro array input data and corresponding GO terms of selected gene groups. Genes are clustered into a grid where each pixel corresponds to a cluster of co-regulated genes that are the same in every GEDI map within a condition. The colors relate to high (red) or low (blue) expression and allow one to identify genes that are, for example, upregulated in one condition over time but not the other (highlighted regions). This allows the visualization of these gene clusters over time and simultaneously over multiple conditions. (Comparison of gene expression levels for target genes: alpha level p < 0.05, one- way ANOVA with Tukey post hoc test).

Next, the expression data was clustered using k-means into 10 partitions where the genes in each resulting cluster were subjected to GO term analysis (Fig. S4a–d). Genes associated with cell cycle were strongly expressed in fibroblasts and to some extend also in early reprogrammed hFL1s, whereas all other reprogramming groups from day 5 do not any longer express these genes (Fig. S4c). This is in accordance to previous reports that show a rapid initiation of conversion upon delivery of reprogramming factors and that conversion does not require proliferation^24^,^31^. However, in contrast we did observe significant intermediate expression of *NESTIN* after 2 days of transgene expression, which declines over time (Fig. 4c). The expression of *NESTIN* was somewhat un-expected as direct conversion generally is thought of as a process with no proliferating stem cell intermediate. However, just recently a single cell transcriptome timeocurse analysis of induced neurons from mouse fibroblasts using the same conversion genes as in this study also reports a transient *Nestin* expression in a population of cells^32^, supporting our finding that the iNs go thorugh a *NESTIN* expressing state.

In line with this observation, *DCX* expression is significantly increased at day 7 of conversion and this strong increase is restricted to the experimental groups of dualSMADi and 6 compounds (Fig. 4d). On day 7, only 6 compounds induced a strong, intermediate expression of microRNA9 (MiR9), which was reduced to similar level of dualSmad group at day 12 (Fig. 4a). MiR9 is one of the most highly expressed microRNAs in the developing and adult vertebrate brain and has been implicated as a driver of neural conversion^33^,^34^. In addition, MiR124 expression is intermediately increased across all groups at day 7 of conversion, which is reduced by day 12 (Fig. 4b). Furthermore, fibroblasts and converting cells on day 2 of transgene expression revealed similar levels of REST expression, which was significantly reduced by day 7 when treating the converting cells with either dualSmad or 6 compounds (Fig. 4e).

In order to identify dynamic transcriptional changes, we then compared the four different conditions more in detail using the gene expression dynamics inspector (GEDI), which allows the visualization of the same gene expression cluster (depicted as a pixel in the map) through time course experiments over several different conditions (Fig. 4f,g; S5a–e). We found that although the expression profile at day 12 was very similar between the groups, there were differing gene expression dynamics in the 6 compound group. For example, a GO term analysis of one group of genes that peaked specifically at day 7 in the 6 compound group showed that this group contained genes associated with “neuron projection development”, “synaptogenesis” and “axon/axonogenesis”, indicating the importance of these particular genes to be transiently expressed during the reprogramming process. Furthermore, genes associated with the GO term “transcription repressor activity” and “response to oxidative stress” was highly expressed in the 6 compound condition (Fig. 4f). Interestingly, among the most strongly differentially expressed genes at day 2 ESM were CXCL5/6, indicative of increased cytokine- cytokine receptor interaction and chemokine signaling in 6 compound group compared to dualSMADi or other experimental groups (Fig. S6). We also identified signaling pathways from the KEGG database and found that genes associated to Wnt signaling and axon guidance are expressed among both groups along the time line of neuronal reprogramming (Fig. S5d).

## Discussion

The use of small molecules to enhance efficiency of reprogramming, reduce the number of genes required for reprogramming as well as to gain further mechanistic insight into the reprogramming process has successfully been applied in somatic cell reprogramming to pluripotency^11^,^12^,^13^,^14^,^18^,^19^,^20^,^35^,^36^. In direct neuronal conversion of human fibroblasts, chemical inhibition of Gsk3β by CHIR99021 combined with inhibition of the SMAD pathway using activin- like kinase 5 (Alk-5) inhibitor together with noggin, has been shown to greatly increase neuronal purity as well as conversion efficiency^22^,^27^. Recently, it has been shown that mouse and human fibroblasts can be directly reprogramming to induced neurons using forskolin, ISX9, CHIR99021, SB431542 and I-BET151^25^ or using valproic acid, CHIR99021, Repsox, forskolin, SP600125, GO6983 and Y-27632, respectively^24^. In addition, it has been shown that LDN193189, SB431542, TTNPB, Tzv, CHIR99021, valproic acid, DAPT, SAG and Purmo reprograms human astroglial cells into functional neurons.

In this study, we performed an unbiased screen that also identified forskolin as well as an additional set of SMs that enhance reprogramming efficiency, acting by inhibition of Gsk3β (WL12, kenpaullone)- activation of cAMP signaling (WL17, PGE2), SIRT1 activation (EL38, aminoresveratrolsulfate), Src kinase inhibition (KL54, PP2), and HDAC inhibition (EL05, BML210). The specificity of the compounds to activate the particular pathways is supported by the fact that the amongst the top 20 hits several compounds were identified that activate the same pathways (Table S1). Additionally, gene expression analysis confirms activation of the appropriate pathways (Fig. S3g).

We found that the combination of Gsk3β inhibition and cAMP signaling activation yielded strongly increased conversion efficiencies. In the presence of additional regulators such as either a HDAC inhibitor, a Src kinase inhibitor or a SIRT1 activator the neuronal purity was also increased.

It has been shown that the usage of HDAC inhibitors facilitates reprogramming to pluripotency^11^,^12^, that pan- Src kinase family inhibitors allow for omitting of Sox2 in generation of iPS cells and activation of sirtuins complement for Klf4 expression^19^,^20^. Also cAMP signaling activators such as forskolin and prostaglandin E2 (PGE2) have been shown to play a crucial role in generating chemically induced pluripotent stem (CiPS) cells^21^ and have been proven to be beneficial for the generation of induced cholinergic neurons via direct conversion from human fibroblasts^23^. Thus, elevated intracellular cAMP levels mediated by forskolin as well as inhibition of Gsk3β and inhibition of activin A receptor type II-like kinase (Alk) 2, 3 and 5 appear to be commonalities facilitating neuronal reprogramming^22^,^24^,^25^,^27^,^37^. Thus, we provide further evidence that modulation of some key signaling pathways promotes both induction of pluripotency and direct neuronal conversion.

Several of the compounds identified in our screen have also previously been reported to be involved in neuronal survival and protection. For example, SIRT1 activators such as resveratrol have also been shown to exert neuroprotective function in rodent models of ALS and Alzheimer’s Disease (AD) and to promote neuronal survival^38^. Furthermore, SIRT1 activators are generally associated with the treatment of age- related diseases^39^ and the Gsk3β inhibitor kenpaullone has also been shown to improve motor neuron survival in an amyotrophic lateral sclerosis (ALS) disease background^17^.

Our genome wide expression analysis showed that the addition of the identified compounds resulted in earlier expression of genes associated with neuron projection development, synaptogenesis and axon/axonogenesis and also genes associated with high transcriptional activity and mediation of cellular stress, suggesting that the reprogramming kinetics is important for final outcome. This is interesting in light of a current report demonstrating that addition of Bcl-2 resulted in faster neuronal differentiation and higher yield during direct neural conversion and that anti-oxidative treatments leads to an improved glial-to-neuron conversion *in vitro* and *in vivo*^40^.

Taken together, our data suggest that triggering multiple signaling cascades that have previously been implicated in enhancing reprogramming of iPSCs and hiNs, together with molecules involved in neuroprotection and survival simultaneously, accelerates direct neuronal reprograming process. The exact mechanisms, and how the interplay among these pathways converge to potentiate cell fate conversion remains to be studied.

## Methods

### Cell origin and culture procedures

Human Fetal Lung Fibroblasts (hFL1) (ATCC- CCL- 153) were purchased from the American Type Culture Collection (ATCC). hFL1 cells were expanded in MEF medium [DMEM (Gibco) supplemented with 100 mg/mL Penincilin/Streptomycin (Sigma), 2 nM L-Glutamine (Sigma), and 10% FBS (Biosera)] and grown at 37 °C in 5% CO_2_. Passage 15 hFL1 cells were used for all experiments. hFL1 cells were dissociated (0.25% Trypsin (Sigma)) and plated in MEF medium at a density of 4 000 cells/cm^2^ in 96-well plates (Nunc), previously coated with 0.1% Gelatin.

### Viral vectors

Doxycycline- regulated lentiviral vectors (LVs) carrying the mouse open reading frames (ORFs) for Ascl1, Brn2 and Myt1L (ABM) were used and have been described in detail elsewhere^8^. A separate LV expressing the TET-ON transactivator (FuW.rtTA-SM2, Addgene) was always co- transduced to activate transgene expression of ABM upon doxycylin administration.

### Generation of hiNs and assay definition

hiNs from hFL1 were generated by transduction of the cells with LVs encoding for the conversion factors ABM (MOI = 5) and co- transduction of the transactivator (FuW.rtTA-SM2, Addgene) (MOI = 10). Transgene expression was initiated by doxycycline (2 μg/mL, Saveen & Werner) administration on day 5 after infection. On day 3 of transgene expression, ABM- transduced (*sample*) as well as untransduced cells (*CNTneg*) received neuronal induction medium (N2B27 + doxycycline 2 μg/mL). For cells of the condition *CNTpos*, neuronal induction medium was supplemented by CHIR99201 (2 μM, Axon), SB431542 (10 μM, R&D Systems), noggin (100 ng/mL, R&D systems), LDN (0.5 μM, Axon), LM4A22 (2 ng/mL, R&D system), GDNF (2 ng/mL, R&D system), NT3 (10 ng/mL, R&D Systems) and db-cAMP (0.5 mM, Sigma). Medium was replaced to ¾ every second- third day and cells were analyzed on day 12 of transgene expression.

### Compound Addition on hiNs

For primary hit identification, individual compounds were added at concentrations 1 μM and 10 μM (Kinase library only 10 μM) to the *sample* condition. For dose response experiments, individual compounds were added in a serial dilution of eleven steps (50 μM – 48.8 nM). Secondary hits were validated using dry- compounds, performing identical dose- response series as described before. All compound addition was performed using automated liquid handling (Agilent Bravo workstation and VWorks 11.4), except for the Wnt- library primary screen and application of compounds in groups (manual addition).

### Immunostaining, Imaging and automated quantifications

Immunohistochemical procedures were performed as has previously been described^6^. An inverted Leica microscope (DFC360 FX + DMI 6000B) was used to capture all images. The total number of DAPI^+^ and MAP2^+^ cells per well was quantified using the Cellomics Array Scan (Array Scan VTI, Thermo Fischer). Using the program “Target activation”, 20 fields (10x magnification) were automatically captured in a spiral fashion (from center to outside), yielding positive cell counts and determination of neuronal purity of cells.

Cytotoxicity of tested compounds was determined by reduced cell number indicated by reduced DAPI^+^ cell counts as well as visual control of each individual wells. Conversion efficiency was determined as described previously^6^ and neuronal purity was determined as the number of MAP2^+^ DAPI^+^ cells out of all DAPI^+^ cells present in the scanned fields at time of analysis.

### Statistical analysis and hit identification

All data are expressed as mean ± the standard deviation. All statistical analyses were conducted using Microsoft Excel or GraphPad Prism 6.0c. An alpha level of p < 0.001 was set for significance when defining assay borders. Neuronal purity of converted neurons among different assay defining conditions was compared using a one-way ANOVA with a Tukey post hoc test, alpha p- level p < 0.001. Assessment of assay condition separation was done calculating the Z- Factor as described elsewhere^28^.

Compounds were identified as hits when inducing a neuronal purity relative to *CNTpos* above the set threshold (mean relative neuronal purity of *sample* + 3xSD) over all plates per experiment.

Comparison of cell number, neuronal purity and conversion efficiency of different compound groups to CNTpos was performed by using a one-way ANOVA with a Bonferroni post hoc test, alpha p- level p < 0.05. Comparison of expression levels of target genes from microarray analysis was performed using a one- way ANOVA with Tukey post hoc test, alpha level p < 0.05.

### Microarray and bioinformatic analysis

hFLs were converted as described above. Cell were harvested for RNA- extraction and subsequent microarray analysis on day 2, 5, 7 and 12 of transgene expression, with day 5 corresponding to 2 days exposure to compounds, day 7 to 4 days in compounds and day 12 to 9 days in compounds. Unconverted fibroblasts in expansion medium were used as untreated reference control. Total RNA of converted cells as well as unconverted cells was collected using the Qiagen MicroKit according to the manufacturers guidelines.

Global gene expression was measured by microarray utilizing the Affymetrix HG-U133 Plus 2.0 array using standard protocols (KFB, Germany). Raw data was RMA normalized using the affy package for *R*, and differentially expressed genes were identified using LIMMA^41^ for all pairwise comparisons of samples (Fig. 4 F: FDR e4; Fig. 4 G: FDR e2; Figure S4: FDR e4; Figure S5 C: FDR e8 Fib; Figure S6: FDR e4; Figure S7: FDR e7, and genes were called differential if the false discovery rate (FDR) was less than or equal to 0.01. Heatmaps (shown in the supplementary figures) were made by applying the k-means algorithm to the differentially expressed genes using Genesis^42^. GEDI maps were produced using the Gene Expression Dynamics Inspector^43^ and enriched ontologies and KEGG pathways were found using DAVID^44^. All data have been deposited in the Gene Expression Omnibus under GEO series accession number GSE83896.

## Additional Information

**How to cite this article**: Pfisterer, U. *et al*. Small molecules increase direct neural conversion of human fibroblasts. *Sci. Rep.* **6**, 38290; doi: 10.1038/srep38290 (2016).

**Publisher's note:** Springer Nature remains neutral with regard to jurisdictional claims in published maps and institutional affiliations.

## Supplementary Material

Supplementary Information
