# Supplementary material for: Small molecules increase direct neural conversion of human fibroblasts
Source: Sci Rep. 2016 Dec 5;6:38290. doi: 10.1038/srep38290 (PMC5137010; doi:10.1038/srep38290)
Supplement: Supplementary Information [file srep38290-s1.pdf]

Title:

Small molecules increase direct neural conversion of human fibroblasts

Authors:

Ulrich Pfisterer[1,4], Fredrik Ek[2], Stefan Lang[2,4], Shamit Soneji[2,4], Roger Olsson[2] and Malin Parmar[1,4]

Table S1. Summary of Primary hits based on the induction of a high neuronal purity

	**Compound**	**Target**	**Mode of Action**
WL12	Kenpaullone	GSK3b	Inhibitor
WL16	Resveratrol	Akt & Erk/ GSK3b	Increase/ Decrease
WL17	PGE2	cAMP/PKA	Unknown
WL25	Forskolin	Adenylyl cyclase	Activator
WL56	Flavanone	Transcription of b-catenin/ Tcf responsive genes	Inhibitor
NL19	Diindolylmethane	AHR	Agonist
NL33	Carbacyclin	PPARd	Agonist
EL05	BML-210	HDAC	Inhibitor
EL08	Scriptaid	HDAC	Inhibitor
EL19	M-344	HDAC	Inhibitor
EL21	BML-266	SIRT2	Inhibitor
EL36	CI-994	HDAC	Inhibitor
KL02	PP1	Src Family	Inhibitor
KL36	Hypericin	PKC	Inhibitor
KL43	KN-62	CaMK II	Inhibitor
KL54	PP2	Src Family	Inhibitor
KL55	ZM 336372	cRAF	Inhibitor
KL58	GW 5074		
KL70	Indirubin		

*Compound EL38 was selected based on the induction of a strong neuronal morphology

Table S2. Summary table of compounds confirmed as dry- powder as well as dry powder compounds tested in groups

a

Compound	Compound Name	Conc.opt (library compound)	Conc.opt (dry compound)
WL12	Kenpaullone	6.25µM	781nM
WL17	PGE2	12.5µM	12.5µM
WL25	Forskolin	25µM	25/ 12.5µM
EL05	BML 210	6.25µM	781nM
EL38	Aminoresveratrol Sulfate	25µM	3.125µM
KL54	PP2	781nM	781nM

b

Group 1	Group 2	Group 3	Group 4	Group 5	Group 6	Concentration	Pathway/ Target
WL12	WL12	WL12		WL12	WL12	781nM	GSK3 inhibitor
WL17	WL17	WL17		WL17		12.5µM	cAMP/PKA
WL25	WL25	WL25			WL25	12.5µM	Adenylyl cyclase Activator
EL05	EL05		EL05		EL05	781nM	HDAC Inhibitor
EL38	EL38		EL38	EL38		3.125µM	SIRT1 Activator
KL54	KL54		KL54			781nM	Src Family
	+ SMs CNTpos						GSK3 inhibitor/ Smad inhibitor

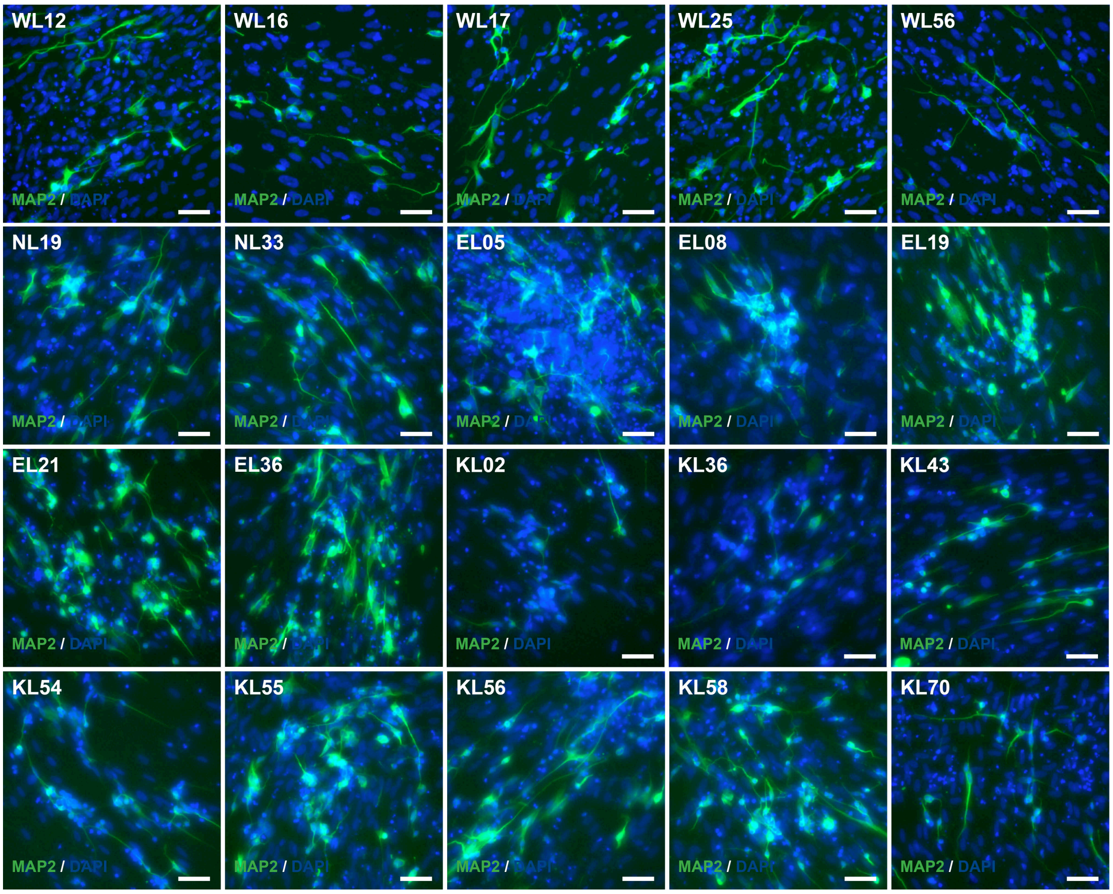

Figure S1. Primary hit compound identification. Representative fluorescence images of selected primary hit compounds corresponding to Figure 1g (Scale bars 50μm).

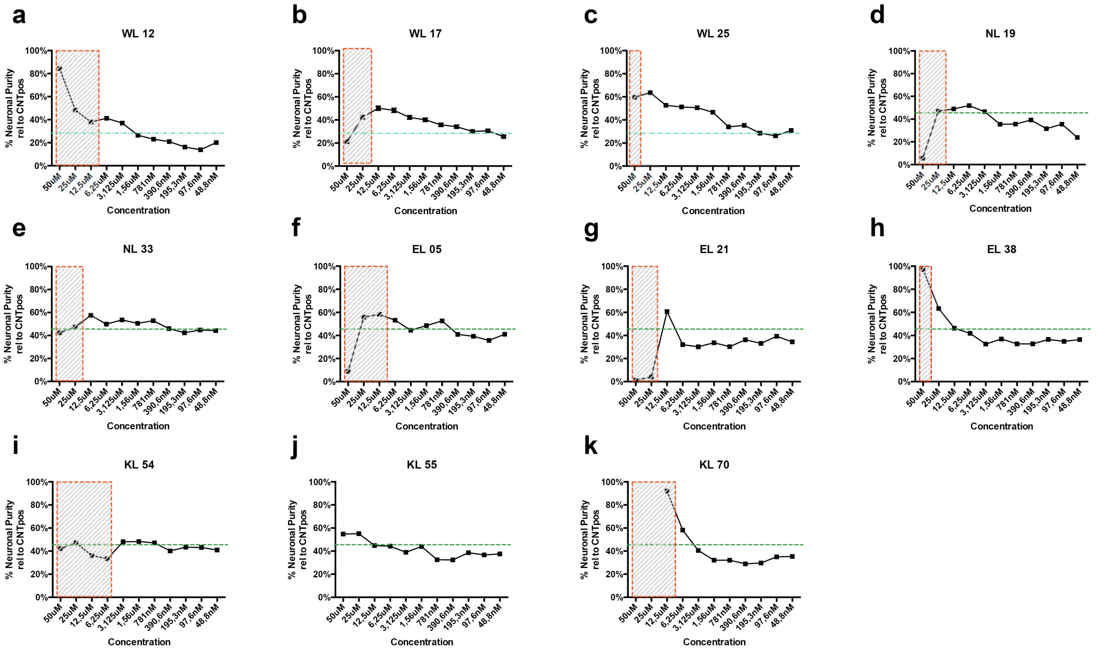

Figure S2. Primary compound hit validation. a- k. Dose- response retesting of primary hit compounds. All compounds were retested at a series of eleven different concentrations (range: 50μM – 48.8nM) and analyzed based on the relative neuronal purity induced as well as cytotoxicity (indicated by grey fields). Primary hits were counted as validated when a dose- dependent increase in relative neuronal purity above the significance threshold (dotted green lines) was observed.

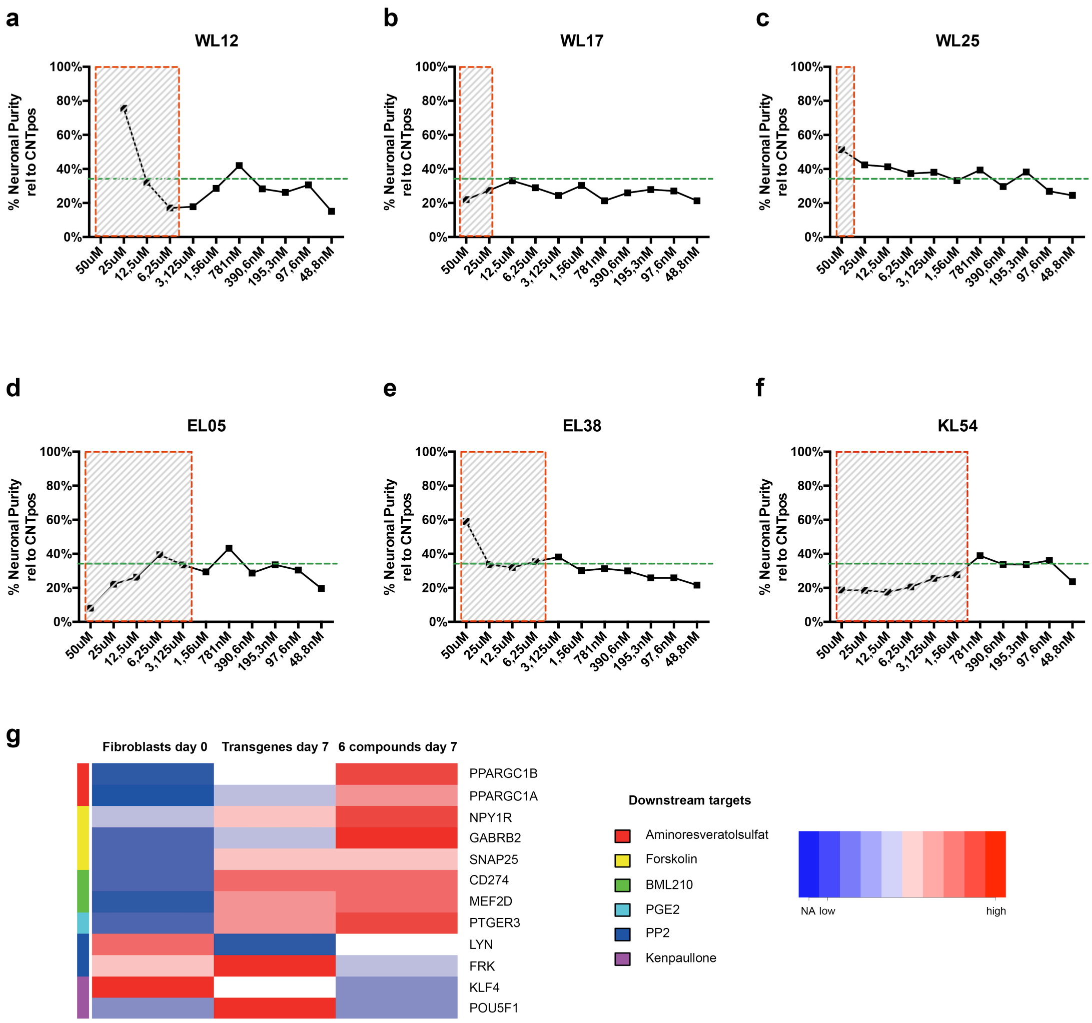

Figure S3. Figure S3. Refinement of dose- response testing and validation of pathway activation. a- f. Dose- response retesting of validated primary hits using corresponding dry compounds. All dry compounds were tested and analyzed identically as for the primary compound hit validation. In a, d and e, usage of dry- compounds shifts concentration optimum to a lower concentration. (Dotted green line: average sample + 3xSD). g. validation of downstream pathway activation in response to addition of the six compounds.

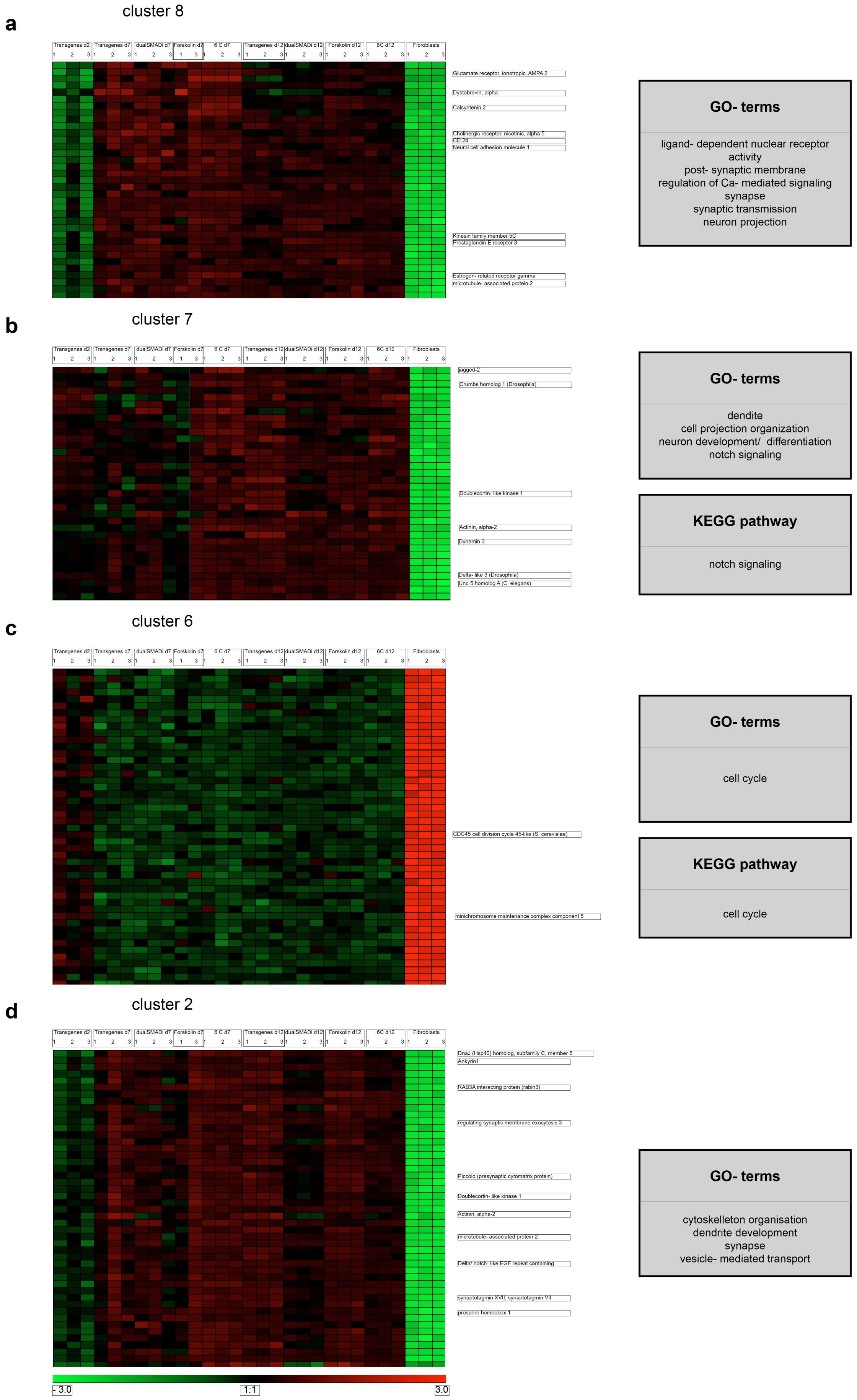
a
cluster 8
Transgenes d2
Transgenes d7
dualSMADi d7
Forskolin d7
6 C d7
Transgenes d12
dualSMADi d12
Forskolin d12
6C d12
Fibroblasts
Glutamate receptor, ionotropic, AMPA 2
Dystobrevin, alpha
Calsyntenin 2
Cholinergic receptor, nicotinic, alpha 5
CD 24
Neural cell adhesion molecule 1
Kinesin family member 5C
Prostaglandin E receptor 3
Estrogen- related receptor gamma
microtubule- associated protein 2
GO- terms
ligand- dependent nuclear receptor activity
post- synaptic membrane
regulation of Ca- mediated signaling
synapse
synaptic transmission
neuron projection
b
cluster 7
jagged-2
Crumbs homolog 1 (Drosophila)
Doublecortin- like kinase 1
Actinin, alpha-2
Dynamin 3
Delta- like 3 (Drosophila)
Unc-5 homolog A (C. elegans)
GO- terms
dendrite
cell projection organization
neuron development/ differentiation
notch signaling
KEGG pathway
notch signaling
c
cluster 6
CDC45 cell division cycle 45-like (S. cerevisiae)
minichromosome maintenance complex component 5
GO- terms
cell cycle
KEGG pathway
cell cycle
d
cluster 2
DnaJ (Hsp40) homolog, subfamily C, member 6
Ankyrin1
RAB3A interacting protein (rabin3)
regulating synaptic membrane exocytosis 3
Piccolo (presynaptic cytomatrix protein)
Doublecortin- like kinase 1
Actinin, alpha-2
microtubule- associated protein 2
Delta/ notch- like EGF repeat containing
synaptotagmin XVII; synaptotagmin VII
prospero homeobox 1
GO- terms
cytoskelleton organisation
dendrite development
synapse
vesicle- mediated transport
- 3.0
1:1
3.0

Figure S4. Micro array gene expression analysis and GO Terms. a- d. K-means clustering of micro array data divided all genes into 10 clusters using the software Genesis. GO term analysis using the online tool DAVID resulted in identification of the presented clusters and heatmaps including genes corresponding to relevant GO terms and KEGG pathways.

a

GO- term	Involved genes
neuron projection development<br>synaptogenesis<br>axon/ axonogenesis	L1 cell adhesion molecule<br>calcitonin-related polypeptide alpha<br>dynamin 3<br>glutamate decarboxylase 1 (brain, 67kDa)

c

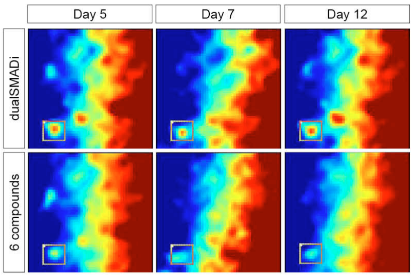

b

GO- term	Involved genes
neurofilament cytoskeleton organ-ization<br>response to oxidative stress<br>transcription repressor activity	ceroid-lipofuscinosis, neuronal 8<br>glutamate-cysteine ligase (catalytic subunit)<br>solute carrier family 23, member 2<br>Sin3A-associated protein<br>inhibitor of DNA binding 2<br>protein inhibitor of activated STAT 4<br>zinc finger protein 148

d

KEGG pathways
Axon guidance
Wnt pathway

e

GO- term	Involved genes
neuron differentiation<br>ion transport<br>neurotransmitter transport<br>transmission of nerve impuls<br>synapse<br>small GTPase binding	ankyrin repeat and sterile alpha motif domain containing 1B<br>ATPase, Ca++ transporting, plasma membrane 2<br>ATPase, Na+/K+ transporting, alpha 3 polypeptide<br>brevican<br>ceroid-lipofuscinosis, neuronal 8<br>dishevelled associated activator of morphogenesis 1<br>FERM, RhoGEF and pleckstrin domain protein 2<br>FYVE, RhoGEF and PH domain containing 4<br>galactose-3-O-sulfotransferase 1<br>gamma-aminobutyric acid (GABA) A receptor, beta 3<br>Kv channel interacting protein 3, calsenilin<br>LIM homeobox 2<br>LIM homeobox 6<br>one cut homeobox 2<br>potassium inwardly-rectifying channel, subfamily J, member 5<br>prostaglandin E receptor 3<br>protein tyrosine phosphatase, non-receptor type 11<br>protocadherin beta 10/ beta 9<br>similar to Rho-associated, coiled-coil containing protein kinase 1<br>solute carrier family 17, member 7<br>solute carrier family 6 (neurotransmitter transporter, dopamine), member 3<br>synaptotagmin XVII; synaptotagmin VII<br>unc-13 homolog A<br>unc-5 homolog A

Figure S5. GEDI analysis using micro array input data. a, b. GO terms and corresponding genes of GEDI analysis in figure 7 d and e, respectively. c, e. Additional GEDI analysis and corresponding GO terms as well as genes. d. Identified KEGG pathways from GEDI analysis in c.

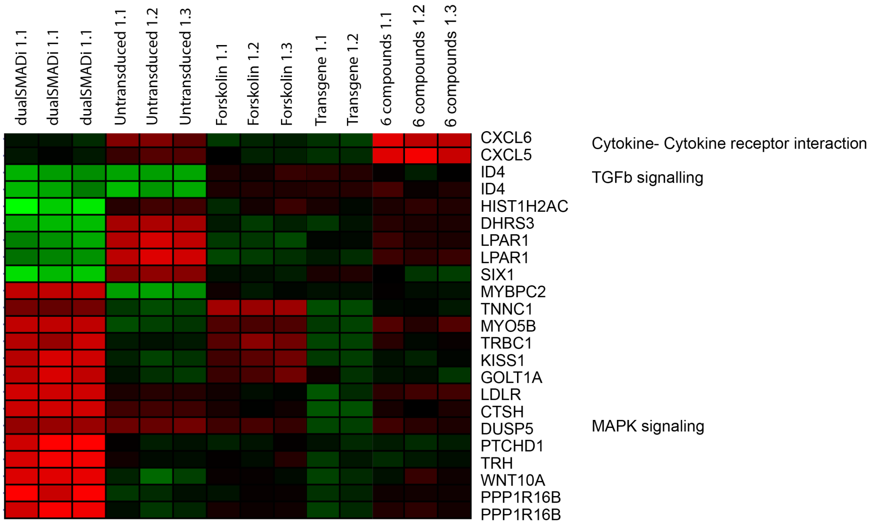

Figure S6. Microarray analysis comparing genes significantly differentially expressed in dualSMADi and 6 compound groups at day 2 ESM. Immediate, early significant gene expression alterations comparing dualSMADi and 6 compound group identified strong expression of CXCL5/6 in 6 compounds indicating increased mediation of cytokine- cytokine receptor interaction and chemokine signaling. Absent expression of ID4 in dualSMADi group and low expression in 6 compound group indicates regulation of Tgf-β signaling. Strong expression of DUSP5 in dualSMADi group and reduced expression in 6 compound group indicates differential regulation of MAPK signaling.
